# Effect of Storage Conditions on Efficacy of Poly(ethylenimine)-Alumina CO_2_ Sorbents

**DOI:** 10.1021/acsomega.5c10742

**Published:** 2026-02-19

**Authors:** Yoseph A. Guta, Iman Nezam, Juliana Carneiro, Samantha Waters, Enerelt Burentugs, Carsten Sievers, Christopher W. Jones

**Affiliations:** School of Chemical and Biomolecular Engineering, 1372 Georgia Institute of Technology, Atlanta, Georgia 30332, United States

## Abstract

Solid amine sorbents are one of the primary components of DAC technologies that allow for the removal of ultradilute CO_2_ from the atmosphere. A main drawback in the implementation of solid amine sorbents in industrial-scale DAC applications is their instability under certain operational or storage conditions over an extended period. In this work, the effect of storage temperature and gas composition in the storage headspace on the long-term stability of a poly­(ethylenimine)-alumina (PEI/γ-Al_2_O_3_) sorbent is explored. PEI/γ-Al_2_O_3_ sorbents with 70 and 100% pore filling are aged under varying gases (N_2_, O_2_, Ar, 0.04% CO_2_–N_2_, CO_2_, and ambient air) in an oven (40 °C), at common ambient indoor temperature conditions (23 °C), or in a freezer (−4 °C). The CO_2_ sorption capacity, as measured by thermogravimetric analysis (TGA), along with FTIR spectra of the fresh and aged sorbents, reveal that at 23 and −4 °C, storage under ambient air or inert gas (Ar) provides reasonable long-term stability, with <13% degradation over 12 and 5 months of storage. Interestingly, with storage at 40 °C, similar levels of deactivation were observed under pure O_2_ and N_2_ after 4 months of storage, which suggests that nonoxidative thermal reactions can occur under prolonged storage conditions under N_2_. In contrast, with storage under CO_2_, sorbent degradation is substantially suppressed compared to storage under N_2_, ambient air, O_2_, or Ar, yielding sorbents with no observable loss in capacity after 2 months, compared to a 66, 63, and 62% loss under N_2_, ambient air, and N_2_ in the same period at 40 °C, respectively. Overall, these findings provide guidance for practical amine sorbent storage in academic or industrial settings where amine sorbents are used for carbon capture.

## Graphical abstract

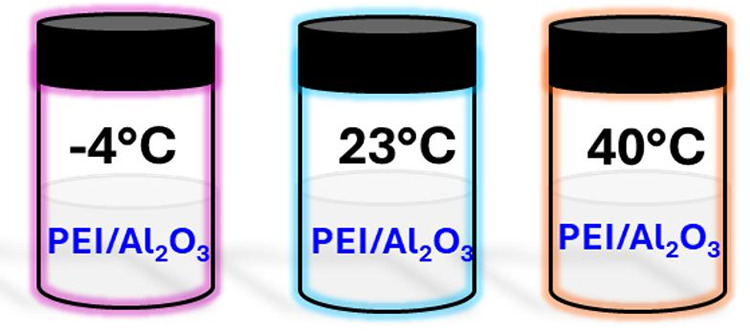

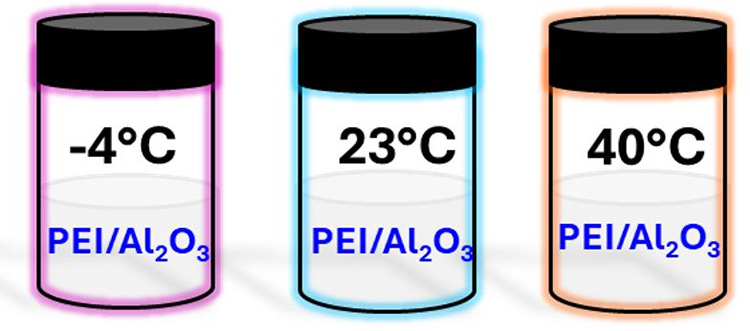

## Introduction

The impact of global warming due to decades of CO_2_ emissions into the atmosphere has become severe in recent years.^1^ Negative emission technologies (NETs) are one of the main mitigation techniques for the removal of CO_2_ and the reduction of the continuously increasing global temperature.^1–4^ Direct air capture (DAC), which selectively extracts CO_2_ from the atmosphere, can be a negative emissions technology when the produced CO_2_ is stored geologically. In addition to enabling CO_2_ storage, DAC also provides an opportunity for the utilization of CO_2_ in the production of value-added products.^5–8^ Currently, there are several DAC plants worldwide contributing to the removal of hundreds to thousands of tons of CO_2_ annually.^2,3,5,9^ Considering the rapidly growing concentration of CO_2_ (∼420 ppm) in the atmosphere and the need for fast, large-scale removal, there is significant potential for DAC technology deployment if the costs can be reduced via research and development.^2,3^

Recently, DAC technologies utilizing solid amine sorbents have shown great promise on the path to industrial-scale implementation and commercialization.^2,5,10^ Solid amine sorbents are suitable for removing CO_2_ from the atmosphere due to the unique acid–base interactions of CO_2_ and amine species that allow for high CO_2_ adsorption capacity (1–2 mmol CO_2_/g sorbent) and fast adsorption rates in a variety of climates and weather conditions.^5,11^

Despite their advantages, solid amine sorbents experience performance degradation during temperature-swing adsorption (TSA) cycles. DAC systems are required to operate for many thousands of adsorption/desorption cycles to be economically viable, energy efficient, and environmentally sustainable.^2,12^ Hence, sorbent lifetime is one of the key parameters affecting the economics and deployment of DAC technologies. Sorbents with longer lifetimes can help alleviate some of the challenges associated with the implementation of DAC^12–14^ or related BECCS (bioenergy with carbon capture and storage)^15^ systems, aiding in the reduction of the currently high capital and operational costs associated with frequent sorbent replacement and the large size of most DAC systems.^2,13,16^ Accordingly, studies focusing on the long-term stability of solid amine sorbents are essential to gain further insight into the different components and parameters affecting their performance.

The degradation of amine sorbents by oxidation,^17–26^ urea formation,^26–29^ or other means^21,27,30^ during TSA cycling is being increasingly studied. However, thus far, there have been only a few studies assessing the degradation of solid amine sorbents under long-term storage conditions. To the best of our knowledge, the first reported study exploring the long-term aging of solid amine sorbents is by Vue et al. They investigated the retained CO_2_ adsorption capacity of a 30 wt % tetraethylenepentamine (TEPA/SiO_2_) sorbent stored under air at ambient temperature for 15 months. Their results indicate that the sorbent lost 48% of its initial CO_2_ adsorption capacity.^31^ In another work, Rosu et al.^24^ investigated the CO_2_ adsorption performance of aged linear poly­(propylenimine) (LPPI)/SBA-15 sorbents and aged LPPI oligomers with varying number-average molecular weight (*M*_n_) (700, 6700, 36000) physically impregnated into fresh SBA-15 supports under air (21% O_2_/balance N_2_). The study reports similar CO_2_ adsorption capacity for both aged LPPI/SBA-15 sorbent and aged LPPIs physically impregnated into fresh SBA-15, with ∼20% loss in capacity after ∼1.5 and 2 years of storage, respectively.^24^ In another study, Priyadarshini et al.^32^ assessed the long-term stability of poly­(ethylenimine) (PEI)/γ-Al_2_O_3_ and (TEPA)/γ-Al_2_O_3_ sorbents considered for ambient (∼25 °C) and subambient (−20 °C) DAC deployment conditions. The study reports a loss of ∼15 to 20% in CO_2_ uptake for both sorbents after 6 months of storage.^32^ The work by Rosu et al.^24^ suggests that PPI-based sorbents display better long-term stability compared to the PEI-based sorbents investigated by Priyadarshini et al.,^32^ further supporting the work by Pang et al.^33,34^ showing enhanced oxidative stability under accelerated oxidative conditions for PPI-based sorbents compared to PEI-based sorbents.

Recently, a study by Al-Absi et al.^35^ examined the impact of extended aging on the stability and chemical structure of a PM01/MSF sorbent, where linear poly­(ethylamine) (LPEI) was *in situ* polymerized into mesoporous silica foam (MSF). After ∼3 years of extended aging, only ∼12% loss in CO_2_ uptake, and slightly lower surface area, pore volume, and organic loading were reported.^35^ In comparison to the above reports, the PM01­(LPEI)/MSF sorbent^35^ showed enhanced long-term stability over the PPI^24^ and PEI or TEPA-based^24^ sorbents studied previously. Many factors may contribute to the altered stability, including differences in sorbent synthesis method (class 3, LPEI synthesis), support type (silica vs alumina vs MOF, etc.), and aging conditions (storage temperature and gas mixture).^24,32,35^ The above reports highlight the varying long-term stability of amine-based sorbents, emphasizing the need for comprehensive, long-duration studies investigating the impact of various atmospheric components and storage conditions on the durability of amine-based DAC sorbents in storage. Long-term stability (storage) data enable identification of ideal sorbent storage conditions that minimize sorbent degradation and maintain CO_2_ adsorption capacity to produce consistent performance over an extended period in academic or industrial research settings, as amine sorbents are susceptible to various environmental components.

Solid amine sorbent instability has mostly been investigated under simulated operational conditions and therefore has been mostly attributed to the susceptibility of amine species (aminopolymers) to oxidation, which subsequently leads to degradation reactions.^25,27,30,36,37^ The presence of O_2_ in a much higher concentration (∼21%) compared to other reactive species (such as CO_2_ and H_2_O) in the atmosphere has led authors to focus most studies on solid amine sorbent stability on the role of O_2_ in the degradation process.^23,25,36,38–40^ Recent studies, however, show that CO_2_ and H_2_O can substantially influence oxidative degradation by catalyzing specific degradation reactions and/or increasing amine (aminopolymer) mobility, allowing for altered O_2_ diffusion.^20,21,41^ Similarly, trace metal impurities in aminopolymers or support materials (Al_2_O_3_, SBA-15, or SiO_2_) also contribute to sorbent instability by promoting radical species formation and enhancing oxidative and CO_2_-induced degradation reactions.^17,20,42^ These collective findings on the influence of the nature of the support, the presence of H_2_O, CO_2_, and other impurities, as well as temperature on sorbent stability show the complexity of the array of degradation reactions that can occur.

While all the above studies provide important insights into solid amine sorbent stability and degradation mechanisms, studies of extended aging of these sorbents are necessary to predict the lifetime of the sorbent, as well as identify conditions for more effective storage of sorbent inventories. To that end, this work reports an investigation of the long-term stability of a solid amine sorbent built from the most important and scalable amine polymer currently used in DAC sorbent formulations, branched, low molecular weight PEI. Specifically, this study explores the effects of temperature at ∼−4, ∼23, and 40 °C and head space storage gases on the long-term stability of 70 and 100% pore fill PEI/γ-Al_2_O_3_ sorbents.

These specific temperatures represent (i) the ambient temperatures of sorbent storage conditions at different locations around the world where temperature-controlled indoor storage conditions might be absent, and (ii) the adsorption temperatures during the CO_2_ capture phase/step of realistic DAC systems in different regions of the world. In realistic DAC systems, the adsorption phase/step accounts for ∼2/3 of the process time, making the study of the long-term stability of these sorbents at these conditions relevant in predicting the long-term stability of a sorbent, specifically, the stability at the adsorption temperatures.

## Experimental and Material Characterization

### Sample Preparation

Branched poly­(ethylenimine) (PEI) 800 g/mol (weight-average molecular weight) purchased from Sigma-Aldrich, and a commercially available γ-Al_2_O_3_ (mesoporous powder) were used in this study. Sorbents comprising PEI filling 70 or 100% of the pore space of the alumina supports were stored in glass vials at three different temperatures (∼−4, ∼23, and 40 °C), under varying head space gas composition, including N_2_ (Airgas UHP 99.999%), humidified N_2_ (Airgas UHP 99.999%), O_2_, Ar (AR UHP300), 0.04% CO_2_–N_2_, CO_2_ (bone dry grade), and ambient air.

Sorbent synthesis for 70 and 100% pore fill PEI/ γ-Al_2_O_3_: First, 1464 mg (for 70% pore fill) and 1456 mg (for 100% pore fill) of mesoporous γ-Al_2_O_3_ substrate (dried overnight in an oven at 85 °C) were added to a 100 mL round-bottomed flask (RBF) with 30 mL methanol. The γ-Al_2_O_3_/methanol mix was sonicated for 15–20 min until a homogeneous sample was evident (no sediment formation at the bottom). Separately, 936 mg (for 70% pore fill) and 1344 mg (for 100% pore fill) of PEI were weighed in a 20 mL vial and mixed with 10 mL of methanol for 10 min on a stirring plate. Once the γ-Al_2_O_3_/methanol sonication was complete, the PEI/methanol solution was added to the RBF and stirred overnight at 40 °C. Following the overnight stirring, the methanol in the solution was evaporated using rotary evaporation, and the obtained sorbent was dried on a high vacuum line (∼10 mTorr, ∼60 °C) overnight.

### N_2_ Physisorption

Physisorption was used to characterize the pore volume and pore size of the sorbent (PEI/ γ-Al_2_O_3_) and the bare alumina support (γ- Al_2_O_3_) available before and after impregnation. The experiments were performed on a Micromeritics TriStar II 3020 Version 3.02 at −196 °C using 100–150 mg of sorbent. The sorbents were pretreated under vacuum at 60 °C for 10 h. The data were analyzed using the Barrett–Joyner–Halenda (BJH) method. Table S1 presents results derived from the N_2_ adsorption–desorption isotherms for the 70 and 100% pore filling sorbents.

### Thermogravimetric Combustion Analysis

Combustion TGA was used to determine the total organic content (PEI) of the fresh and aged sorbents. Organic combustion analysis was performed on a TGA (Discovery TGA550) by heating the sample from room temperature to 900 °C with a ramp rate of 10 °C/min under air. The weight loss of the sorbent from 200 to 900 °C was taken as the organic content (Figure S1).

### Sample Storage

The containers used in this study for sorbent storage were borosilicate glass vials from VWR with screw caps made of poly­(propylene). For storage under N_2_ or O_2_ gas, the respective gas was introduced into the samples to purge out the ambient air and other weakly sorbed species (e.g., CO_2_ and H_2_O) present in the container using an inlet and outlet line for ∼10 min. Following that, the outlet line was removed and N_2_ or O_2_ gas was flowed into the respective container for ∼20 min. Samples that were aged under ambient air were stored by simply capping the containers after initial sample preparation. For the humidified study, N_2_ gas was flowed through a bubbler with 50% relative humidity at ∼23 °C and then passed into the samples after purging out ambient air in the sample with N_2_ for ∼10 min, with humidified N_2_ flowing for ∼20 min. For samples stored under Ar, the gas was introduced into the sample container by flowing continuously over the sample for ∼30 s and simply capping the container afterward. Finally, for long-term O_2_ leakage tests, the respective gas was introduced into the vials containing the sorbent (primary containers) inside a glovebox containing an N_2_ environment to avoid ambient O_2_ exposure.

### Aging Experiments

After the introduction of the respective gas mixtures, the samples were wrapped completely in aluminum foil and placed in a freezer at ∼−4 °C, in a drawer (at room temperature ∼23 °C), and in an oven (at 40 °C). For long-term O_2_ leakage test, the vials wrapped in aluminum foil were stored in a secondary container filled with N_2_ environment.

### CO_2_ Adsorption Experiment

A thermogravimetric analyzer (TGA) (TA Instruments Q500) was used to investigate the CO_2_ adsorption capacity change as a function of aging temperature and time. CO_2_ adsorption experiments were conducted using a TGA with a 10–20 mg sample that was removed from the storage container and loaded on a 50 μL platinum pan. After removing the desired amount for the CO_2_ adsorption measurement, the vials were restored under their original conditions following the procedure discussed in the sample storage section. To pretreat the sample, He (Airgas UHP300) gas at 90 mL/min was flowed, and the temperature was increased to 100 °C from room temperature at a ramp rate of 10 °C/min and maintained for 1 h. Then, the temperature was lowered to 30 °C at a ramp rate of 10 °C/min. Once the system equilibrated at 30 °C, the gas was switched to 400 ppm of CO_2_ (balance He) at the same flow rate and held for 3 h to allow CO_2_ adsorption. After the 3 h of adsorption, the gas was switched back to He at 90 mL/min and the temperature was increased to 100 °C at a ramp rate of 10 °C/min to desorb the CO_2_. The temperature was held at 100 °C for 1 h to allow complete desorption of CO_2_. At the end of the desorption, the temperature was reduced to 30 °C at a ramp rate of 10 °C/min to cool the instrument. The initial CO_2_ adsorption capacity of the 70% and 100% pore-fill PEI/Al_2_O_3_ sorbents, along with the absolute capacity values for each sample, are shown in Figure S2 and Table S2, respectively. Sorbent deactivation is calculated using eq 1:

1 $$\mathrm{sorbent}\;\mathrm{deactivation}=\frac{{\mathrm{CO}}_{2}\;\mathrm{capacity}\;\mathrm{fresh}-{\mathrm{CO}}_{2}\;\mathrm{capacity}\;\mathrm{deactivated}}{{\mathrm{CO}}_{2}\;\mathrm{capacity}\;\mathrm{fresh}}$$

### Transmission FTIR Experiments

The FTIR experiments were performed using a Thermo Scientific Nicolet IS10 spectrometer to observe changes in the sample’s IR spectra associated with extended storage and aging.

#### Wafer/Pellet Preparation

KBr (90 mg) obtained from an oven at 110 °C was ground using a mortar and pestle before being mixed with 10 mg of the sorbent sample. After mixing, 30 mg of the sample/KBr mix was pressed using a hydraulic press at 1000 psi for 30 s. The weight of the wafers ranged from 22 to 25 mg.

### Spectroscopic Measurements

Before wafers were transferred into a flow-through IR transmission cell and the cell was purged using He (Airgas UHP 99.99%) or N_2_ gas for 20 min. Following that, the wafer was pretreated to 100 °C for 1 h under He or N_2_ across different experiments to remove physisorbed water vapor and other volatile species. The sample spectra were taken after reducing the temperature to 30 °C and stabilizing at 30 °C for 10 min under He. FTIR spectra of pristine 70% and 100% pore fill (PF) PEI/γ-Al_2_O_3_ sorbent at 30 °C are shown in Figure S3.

## Results and Discussion

The long-term effect of amine sorbent exposure to varying storage gas mixtures was explored, varying storage temperature and other factors using 70 and 100% pore fill PEI/γ-Al_2_O_3_ sorbents. The impact on stability was tracked by measuring the change in each sorbent’s CO_2_ adsorption capacity (mmol CO_2_/g sorbent) over time. CO_2_ adsorption measurements were conducted on a TGA by exposing a fraction of each sample to 400 ppm of CO_2_ balance He or N_2_ at 30 °C for 3 h after 1-h pretreatment under He or N_2_ at 100 °C to remove weakly sorbed species. Sorbent deactivation is reported as the percent reduction in the CO_2_ adsorption capacity relative to that of a fresh sorbent after being aged under the specified conditions.

### Effect of Storage Temperature & Support Pore Filling

For ease of storage, many researchers store their sorbent samples under ambient air. To this end, we began our studies probing the impact of storage temperature under air. In parallel, we evaluated samples with 100% of their pore space filled with PEI and compared those to samples that had only 70% of the pore space filled. The latter loading is a common one that seeks to balance sorption kinetics and sorption capacity. Figure 1a shows that storage of the sorbents at common ambient indoor temperature conditions (23 °C) or in a freezer (−4 °C) leads to minimal sorbent degradation over 12 months of storage. Figure 1b illustrates that samples with complete pore filling have less rapid sorbent degradation kinetics, perhaps due to ambient gases having less access to PEI deeply buried within the pore space under 40 °C temperature storage conditions. At the lower two storage temperatures, there was minimal impact of pore filling, likely because the total degradation was very low in all cases (<8% after 12 months) (Figures S4a and S4b).

**Figure 1:**
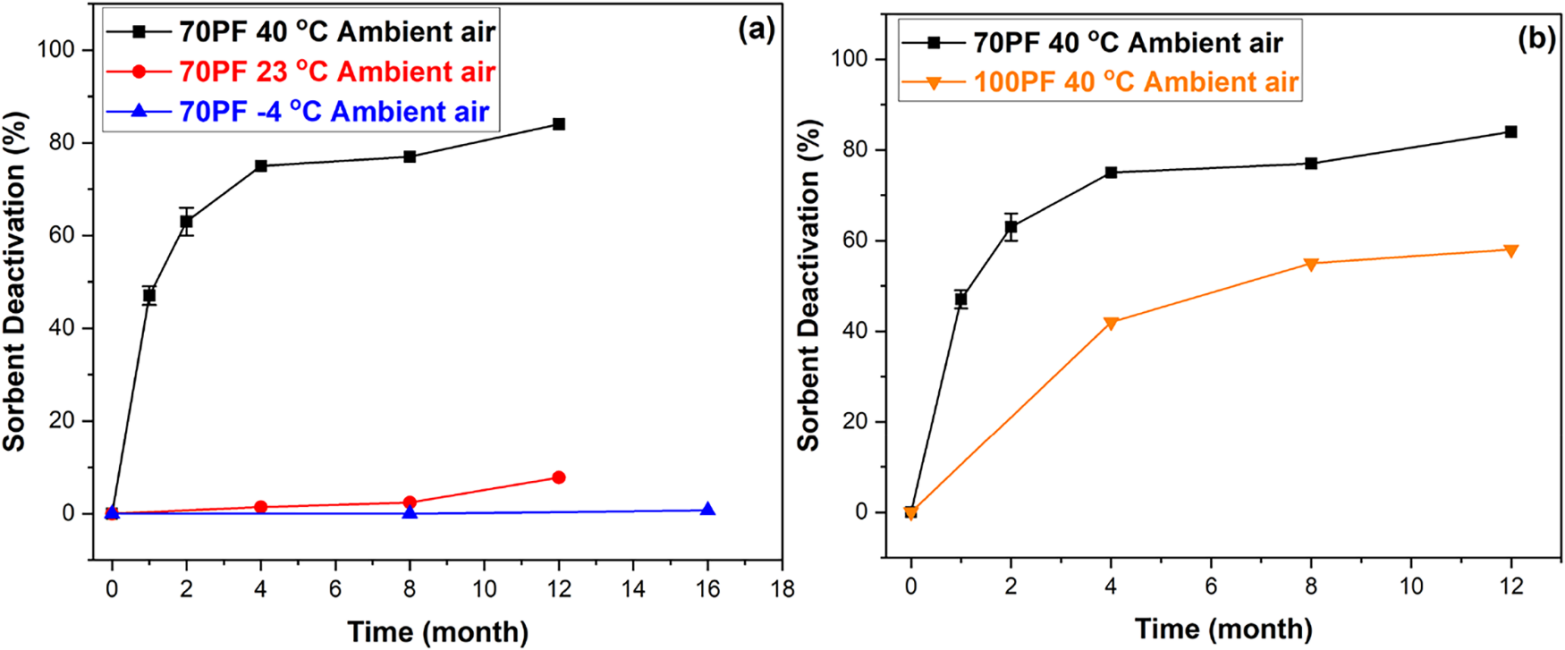
Sorbent deactivation under ambient air for the (a) 70% pore filling (PF) PEI/γ-Al_2_O_3_ sorbent aging at 40 °C, 23 °C, and −4 °C; (b) comparing 70% vs 100% PF PEI/γ-Al_2_O_3_ sorbent aging at 40 °C.

### Effect of Gas Mixtures (Storage Gas)

Knowing that amine sorbents are sensitive to oxidative degradation, many researchers may choose to store their sorbents under inert gases. For this reason, we evaluated the 70% pore-fill PEI/γ-Al_2_O_3_ sorbent after long-term storage using different gas mixtures in the sample vial headspace. At 40 °C, samples stored under N_2_ and O_2_ showed comparable extents of deactivation, being almost fully degraded after only 4 months of storage, while the sample stored under ambient air showed marginally less deactivation (Figure 2a). Furthermore, the FTIR spectra of 4-month-aged sorbents under N_2_ at 40 °C show (Figure 2b) significant formation of amine-related deactivation species such as carbonyl/imine (∼1666 cm^–1^)^20,27,43,44^ bands and newly formed primary amines (∼1602 cm^–1^)^20,21,43^ compared to ambient air. The 1666 cm^–1^ peak under N_2_ can be attributed to imine (C=N) species formation, which is consistent with thermal degradation reactions taking place under long-term storage. The 1666 cm^–1^ peak consist of contributions from primary and secondary imine species at ∼1655 and ∼1675 cm^–1^ peaks, respectively. In addition to the imine species, it is possible that carbonyl species (amides) formed via the reaction of trace O_2_ molecules (most likely introduced during sorbent preparation) and free radicals formed due to thermal stress also contribute to the 1666 cm^–1^ peak. Figure S5 shows the FTIR spectra under O_2_, where the formation of the carbonyl or imine species (∼1666 cm^–1^) and the loss of hydrogen atoms indicated by the negative C–H bending band are significant compared to samples stored under N_2_.

**Figure 2:**
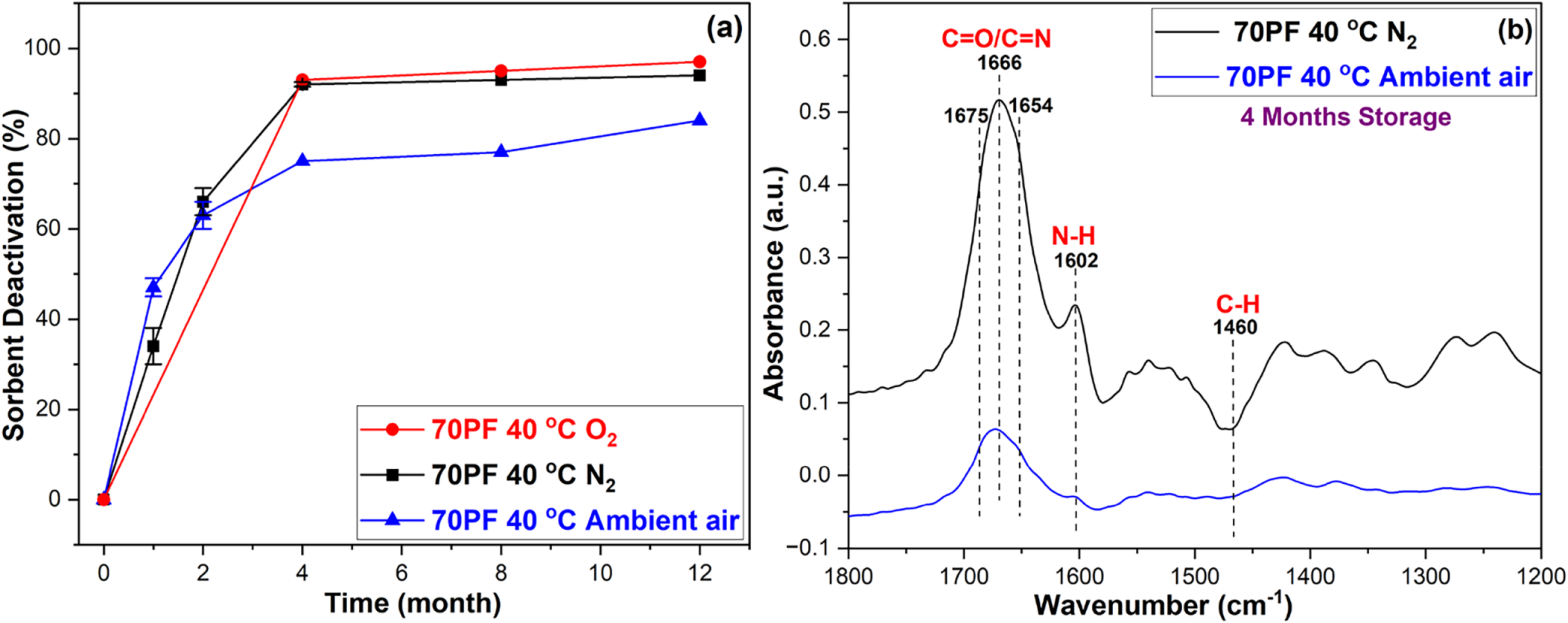
(a) Sorbent deactivation of the 70% pore fill (PF) PEI/γ-Al_2_O_3_ sorbent aged under N_2_, ambient air, and O_2_ at 40 °C; (b) FTIR spectra of 70% pore fill (PF) PEI/γ-Al_2_O_3_ sorbent aged under N_2_ and ambient air at 40 °C

This surprising result – high instability under N_2_ storage (Figure 2a) – was probed by several testable hypotheses. First, we hypothesized that significant nonoxidative thermal reactions were taking place under long-term storage at 40 °C. Second, we hypothesized that treatment with bottled, dry O_2_ or N_2_ may dehumidify the samples to some extent, with water on the sample perhaps offering some protection toward degradation. Third, we hypothesized that CO_2_ from ambient air may protect the samples to some degree under these storage conditions, which may occur by cross-linking PEI when CO_2_ adsorbs as ammonium carbamate. To this end, we compared the degradation of the 70% pore-fill PEI/γ-Al_2_O_3_ sorbent under dry and humidified N_2_ (Figure 3a) and under dry N_2_ and dry CO_2_-containing N_2_ (400 ppm of CO_2_ concentration; Figure 3b). The results (Figure 3a) demonstrate that the presence or absence of humidity does not significantly impact stability under N_2_. The inclusion of a small amount of CO_2_ offers some reduction in degradation (Figure 3b). As the small amount of CO_2_ (∼400 ppm) showed some improvement in stability, we further investigated the sorbent stability under pure CO_2_. The results (Figure 3c) show significant improvement in stability under pure CO_2_ after two months of storage, as indicated by no observable sorbent deactivation in that period. The significant enhancement in stability under pure CO_2_ is most likely due to the PEI cross-linking effect of adsorbed CO_2_ forming ammonium carbamate, which limits chain mobility and minimizes interactions/reactions that accelerate sorbent degradation. A recent study showed that increasing CO_2_ loading above a certain threshold can enhance PEI/Al_2_O_3_ sorbent stability by slowing down radical propagation reactions, as the increased CO_2_ loading promotes faster adsorption kinetics and cross-linking of amine chains as adsorbed CO_2_ species form.^45^ At 23 °C, a pure CO_2_ environment results in no significant degradation after two months of storage, similar to the sorbents stored under pure N_2_ and 0.04% CO_2_–N_2_ (Figure 3d).

**Figure 3:**
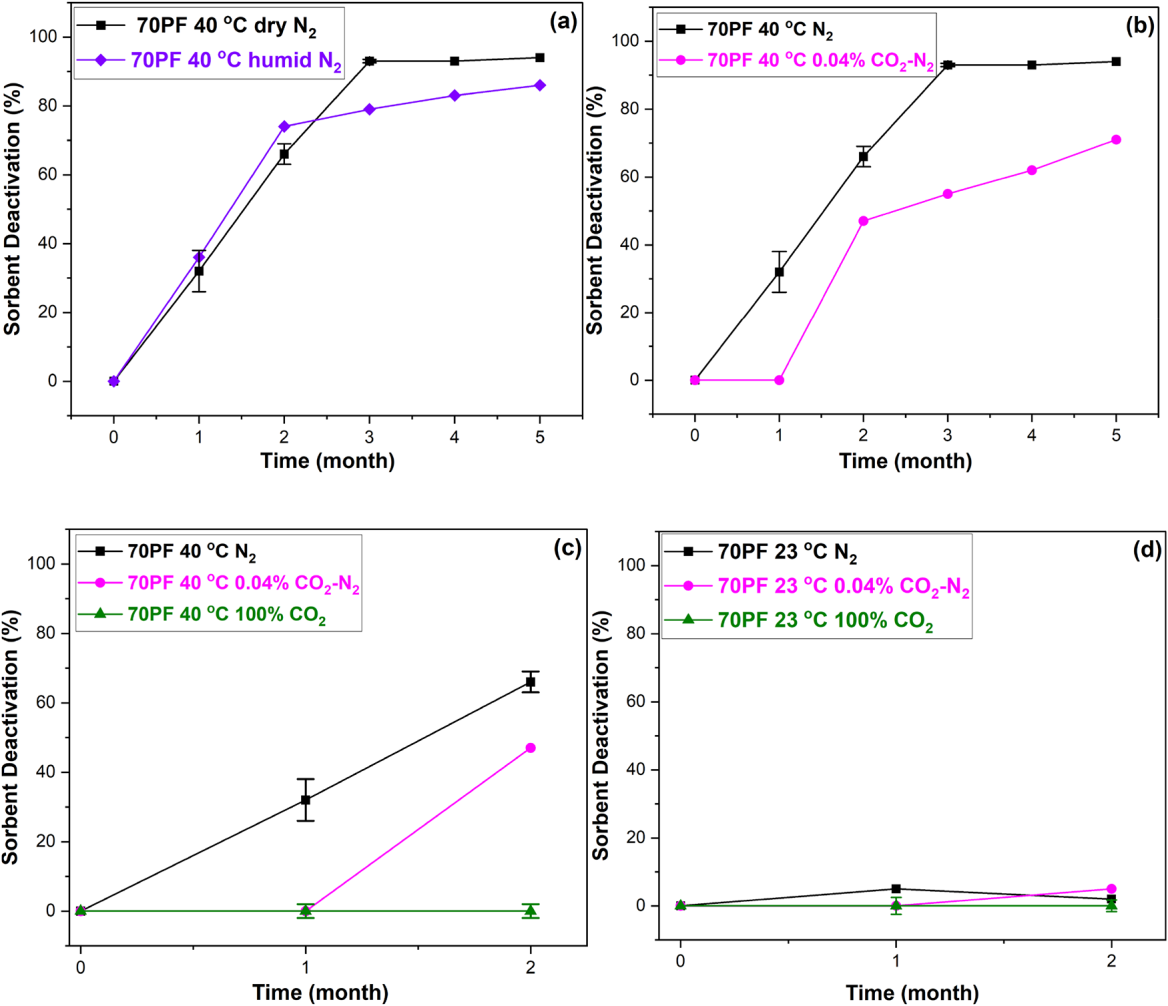
Sorbent deactivation of the 70% pore fill (PF) PEI/γ-Al_2_O_3_ sorbent aged under (a) dry and humidified N_2_ at 40 °C; (b) dry N_2_ and 400 ppm of CO_2_-containing N_2_ at 40 °C; (c) dry N_2_, 400 ppm of CO_2_-containing N_2_, and 100% CO_2_ at 40 °C; (d) dry N_2_, 400 ppm of CO_2_-containing N_2_, and 100% CO_2_ at 23 °C. The error bars for N_2_ at 40 °C and CO_2_ at 23 and 40 °C indicate the standard deviation of deactivation based on three replicate CO_2_ adsorption experiments of three samples stored under the same storage conditions.

To probe the potential for long-term O_2_ leakage from ambient air into the sample vials, vials containing sorbents (70% pore-fill PEI/γ-Al_2_O_3_) filled with N_2_ or air were placed in containers filled with dry N_2_ and were stored in an oven at 40 °C. as noted in the experimental section, the introduction of the respective storage gas into the vials containing the sorbent (primary containers) was conducted in a glovebox containing an N_2_ environment to avoid ambient O_2_ exposure. The stability of the sorbents stored in a secondary container filled with N_2_ was compared with that of sorbents stored in vials exposed to a noninert oven environment (vials placed in an oven without N_2_-filled secondary containers). Furthermore, the temperature inside the secondary container was confirmed to be the same as the oven temperature by placing a thermocouple probe inside the secondary container in a separate experiment. The result in Figure 4a shows similar deactivation for both sorbents stored in a secondary container (N_2_-filled) and without a secondary container after two months of storage. These results suggest that long-term O_2_ leakage is unlikely to be a cause of the substantial sorbent degradation under an N_2_ environment at 40 °C (Figure 2a) and that the degradation under N_2_ is mostly due to nonoxidative thermal degradation reactions. Examples of potential nonoxidative, thermal degradation reaction pathways are shown in Scheme S1.

**Figure 4:**
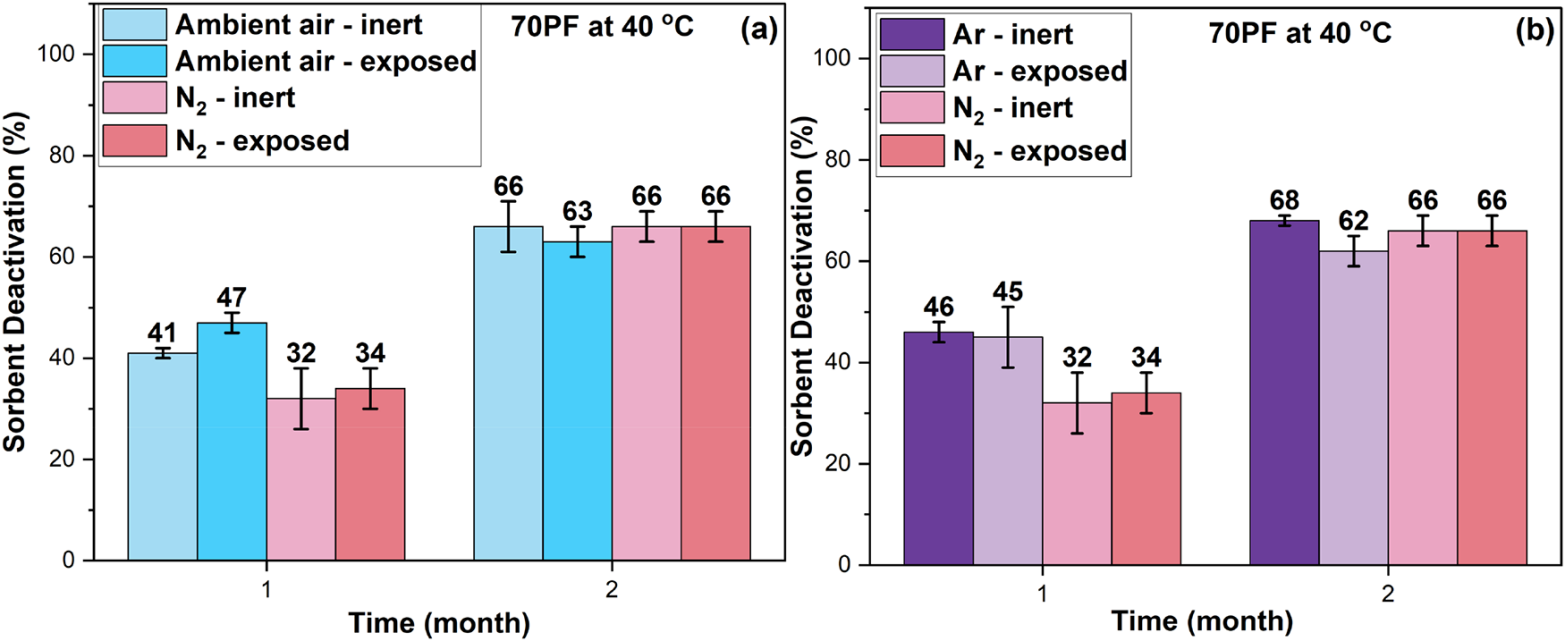
Sorbent deactivation of 70% pore fill (PF) PEI/γ-Al_2_O_3_ sorbent aged under (a) N_2_ and Ambient air within an N_2_-filled secondary container (inert) and without (exposed) at 40 °C; (b) N_2_ and Ar within an N_2_-filled secondary container (inert) and without (exposed) at 40 °C. The error bars indicate the standard deviation of deactivation based on three replicate CO_2_ adsorption experiments of three samples stored under the same storage conditions.

While temperature (thermal effects) contributes to the degradation at 40 °C, we hypothesize that the unexpectedly high degradation under N_2_ can be mainly attributed to the stagnant environments allowing gas-phase products and highly reactive radical fragments that form during the nonoxidative degradation reactions to accumulate and participate in accelerated secondary sorbent degradation reactions. Another secondary factor contributing to the degradation can be trace amounts of O_2_ molecules in the inert environment catalyzing oxidative reactions.

To investigate if the nonoxidative thermal degradation also occurs in another inert gas used for sorbent storage, we performed the same aging experiment of 70% pore-fill PEI/Al_2_O_3_ sorbent under argon at 40 °C. The result illustrated in Figure 4b shows similar deactivation under both N_2_ and Ar storage conditions after 2 months. In addition, Figure 4b also shows that long-term O_2_ leakage contributing to the degradation is not observed under Ar, similar to N_2_ and ambient air storage environments. Overall, the similarity in deactivation between the two inert (N_2_ and Ar) storage conditions supports the occurrence of nonoxidative thermal degradation reactions as the main cause of instability. At 23 °C, the degradation of the sorbent stored under both gases was minimal for the period of the study (5 months) ranging from 6 to 16% (Figure S6).

In comparison to the long-term aging reported in Al-Absi et al. work (<12% deactivation after 3 years),^35^ the sorbent stability observed in this study (<10% deactivation in ∼12 months under ambient air at 23 and −4 °C) is lower. Some of the reasons for the differences in stability could be the sorbent synthesis method and polymer type (LPEI *in situ* polymerization (Al-Absi et al.)^35^ vs physical branched bPEI impregnation (this study)), the support type (MSF silica in Al-Absi et al. vs alumina in this study),^35^ and storage conditions (unspecified (Al-Absi et al.) vs under Ar, N_2_ and ambient air at 23 and −4 °C (this study)). In Al-Absi et al., *in situ* polymerized sorbents (class 3) showed improved stability compared to physically impregnated (class 1) and grafted (class 2) sorbents, which they attributed to strong chemical bonding with the support.^35^ Accordingly, the physically impregnated sorbents used in this study should have less stability compared to *in situ* polymerized sorbents, such as those used in the Al-Absi et al. extended aging study.^35^ Furthermore, as has been reported earlier, the type of support material presents an additional variable that can alter sorbent stability due to differences in physical structure (pore volume, pore diameter, and surface area), acidity of the support, and/or trace metal impurity content.^17,20,42^ Finally, the storage conditions may also be an additional factor in the variation between the two studies; however, due to the absence of data about the storage conditions in the Al-Absi et al. study, a direct comparison is unavailable.

## Summary

The impact of sorbent storage gases and temperature on the long-term stability of 70% and 100% pore fill PEI/γ-Al_2_O_3_ sorbents was investigated. It was demonstrated that sorbents stored at 23 °C and −4 °C exhibited reasonable long-term stability (ambient air: <10% deactivation at 23 °C in ∼12 months and <1% deactivation after ∼16 months). At 40 °C, the results show significant sorbent deactivation (an irreversible process) when stored under O_2_ and N_2_ gases, suggesting important oxidative and thermal degradation reactions under those conditions. On the contrary, sorbent stability was slightly improved under ambient air and 0.04% CO_2_–N_2_ and significantly improved under 100% CO_2_ compared to N_2_ at 40 °C. The significant sorbent instability at 40 °C under N_2_ was also observed with an Ar environment after 2 months of storage. This trend was rationalized by the existence of nonoxidative thermal reactions occurring over the long-term exposure period. The enhanced stability under 100% CO_2_ is attributed to the cross-linking effect of adsorbed CO_2_ species, imparting protection under the conditions used.

This study suggests that sorbent storage under pure CO_2_ offers the potential for long-term stability even at temperatures above the ordinary indoor temperature. Moreover, the minimal sorbent degradation under ambient air at −4 °C indicates aminopolymer sorbents such as PEI/ γ-Al_2_O_3_ might have prolonged lifetimes under subambient DAC cycling conditions.^46^ Limitations of this study include the use of only a single support material and a single amine polymer, and further studies of the long-term stability of amine sorbents are warranted.

## Supplementary Material



## Notes

Pore volume and surface area values of the support and sorbents (70% and 100% Pore-fill PEI/Al_2_O_3_), organic content in each sorbent, CO_2_ adsorption capacity of the pristine sorbents (70% and 100% Pore-fill PEI/Al_2_O_3_), Equation for determining sorbent deactivation, FTIR spectra of the pristine 70% and 100% pore-fill PEI/Al_2_O_3_ sorbents, Sorbent deactivation data under ambient air at −4 and 23 °C, FTIR spectra of the pristine, and deactivated sorbent under O_2_, N_2_ and ambient air at 40 °C, Sorbent deactivation data under N_2_ and Ar at 23 °C, Absolute CO_2_ adsorption capacity values for all samples (PDF)

## †

Evonik, 7001 Hamilton Blvd, Trexlertown, Pennsylvania 18087, United States

## ‡

Columbia University, Department of Chemical Engineering, New York, New York 10027, United States.

## §

University of Texas at Austin, McKetta Department of Chemical Engineering and Texas Materials Institute, Austin, Texas 78712, United States.

## ∥

Center for Catalytic Science and Technology, Department of Chemical and Biomolecular Engineering, University of Delaware, Newark, Delaware 19716, United States.

## References

[1] Rogelj J., Luderer G., Pietzcker R. C., Kriegler E., Schaeffer M., Krey V., Riahi K. (2016). Energy system
transformations for
limiting end-of-century warming to below 1.5 °C (vol 5, pg 519,
2015). Nat. Clim Change.

[2] McQueen N., Gomes K. V., McCormick C., Blumanthal K., Pisciotta M., Wilcox J. (2021). A review of direct
air capture (DAC):
scaling up commercial technologies and innovating for the future. Prog. Energy.

[3] Chowdhury S., Kumar Y., Shrivastava S., Patel S. K., Sangwai J. S. (2023). A Review
on the Recent Scientific and Commercial Progress on the Direct Air
Capture Technology to Manage Atmospheric CO_2_ Concentrations
and Future Perspectives. Energy Fuels.

[4] Sanz-Pérez E. S., Murdock C. R., Didas S. A., Jones C. W. (2016). Direct Capture of
CO_2_ from Ambient Air. Chem. Rev..

[5] Navik R., Wang E. R. Y., Ding X., Qiu K. X., Li J. (2024). Atmospheric
carbon dioxide capture by adsorption on amine-functionalized silica
composites: a review. Environ. Chem. Lett..

[6] Wu Q. J., Liang J., Huang Y. B., Cao R. (2022). Thermo-, Electro-,
and Photocatalytic CO_2_ Conversion to Value- Added Products
over Porous Metal/Covalent Organic Frameworks. Acc. Chem. Res..

[7] Zhou H., Docherty S. R., Phongprueksathat N., Chen Z. X., Bukhtiyarov A. V., Prosvirin I. P., Safonova O. V., Urakawa A., Copéret C., Müller C. R., Fedorov A. (2023). Combining Atomic Layer Deposition
with Surface Organometallic Chemistry to Enhance Atomic-Scale Interactions
and Improve the Activity and Selectivity of Cu-Zn/SiO_2_ Catalysts
for the Hydrogenation of CO_2_ to Methanol. JACS Au.

[8] Cordero-Lanzac T., Berdiell I. C., Airi A., Chung S. H., Mancuso J. L., Redekop E. A., Fabris C., Figueroa-Quintero L., de Miguel J. C. N., Narciso J. (2024). Transitioning
from Methanol
to Olefins (MTO) toward a Tandem CO_2_ Hydrogenation Process:
On the Role and Fate of Heteroatoms (Mg, Si) in MAPO-18 Zeotypes. JACS Au.

[9] Zhu X. C., Xie W. W., Wu J. Y., Miao Y. H., Xiang C. J., Chen C. P., Ge B. Y., Gan Z. Z., Yang F., Zhang M. (2022). Recent advances in direct air capture by adsorption. Chem. Soc. Rev..

[10] Hack J., Maeda N., Meier D. M. (2022). Review
on CO_2_ Capture
Using Amine-Functionalized Materials. Acs Omega.

[11] Kong F. H., Rim G., Song M., Rosu C., Priyadarshini P., Lively R. P., Realff M. J., Jones C. W. (2022). Research needs targeting
direct air capture of carbon dioxide: Material & process performance
characteristics under realistic environmental conditions. Korean J. Chem. Eng..

[12] Azarabadi H., Lackner K. S. (2019). A Sorbent-Focused
Techno-Economic Analysis of Direct
Air Capture. Appl. Energy.

[13] Holmes H. E., Banerjee S., Vallace A., Lively R. P., Jones C. W., Realff M. J. (2024). Tuning sorbent properties
to reduce the cost of direct
air capture. Energy Environ. Sci..

[14] Madhu K., Pauliuk S., Dhathri S., Creutzig F. (2021). Understanding environmental
trade-offs and resource demand of direct air capture technologies
through comparative life-cycle assessment. Nat.
Energy.

[15] Holmes H. E., Lively R. P., Realff M. J. (2021). Defining Targets
for Adsorbent Material
Performance to Enable Viable BECCS Processes. JACS Au.

[16] Dziejarski, B. ; Serafin, J. ; Andersson, K. ; Krzyzynska, R. CO_2_ capture materials: a review of current trends and future challenges. Mater. Today Sustain. 2023, 24 100483 10.1016/j.mtsust.2023.100483.

[17] Yan, C. Y. ; Sayari, A. Spectroscopic investigation into the oxidation of polyethylenimine for CO_2_ capture: Mitigation strategies and mechanism. Chem. Eng. J. 2024, 479 147498 10.1016/j.cej.2023.147498.

[18] Hunter-Sellars E., Kerr J. D., Eshelman H. V., Pollard Z. A., Varni A. J., Sakwa-Novak M. A., Marple M. A. T., Pang S. H. (2024). Oxidation of Supported
Amines for CO_2_ Direct Air Capture: Assessing Impact on
Physical Properties and Mobility via NMR Relaxometry. Macromol. Chem. Phys..

[19] Li S. C., Ceron M. R., Eshelman H. V., Varni A. J., Maiti A., Akhade S., Pang S. H. (2023). Probing
the Kinetic Origin of Varying
Oxidative Stability of Ethyl- vs. Propyl-spaced Amines for Direct
Air Capture. ChemSusChem.

[20] Carneiro J. S. A., Innocenti G., Moon H. J., Guta Y., Proano L., Sievers C., Sakwa-Novak M. A., Ping E. W., Jones C. W. (2023). Insights
into the Oxidative Degradation Mechanism of Solid Amine Sorbents for
CO_2_ Capture from Air: Roles of Atmospheric Water. Angew. Chem. Int. Ed..

[21] Guta Y. A., Carneiro J., Li S., Innocenti G., Pang S. H., Sakwa-Novak M. A., Sievers C., Jones C. W. (2023). Contributions
of CO_2_, O_2_, and H_2_O to the Oxidative
Stability of Solid Amine Direct Air Capture Sorbents at Intermediate
Temperature. ACS Appl. Mater. Interfaces.

[22] Racicot J., Li S. C., Clabaugh A., Hertz C., Akhade S. A., Ping E. W., Pang S. H., Sakwa-Novak M. A. (2022). Volatile
Products of the Autoxidation of Poly­(ethylenimine) in CO_2_ Sorbents. J. Phys. Chem. C.

[23] Nezam I., Xie J. W., Golub K. W., Carneiro J., Olsen K., Ping E. W., Jones C. W., Sakwa-Novak M. A. (2021). Chemical
Kinetics of the Autoxidation of Poly­(ethylenimine) in CO_2_ Sorbents. ACS Sustainable Chem. Eng..

[24] Rosu C., Pang S. H., Sujan A. R., Sakwa-Novak M. A., Ping E. W., Jones C. W. (2020). Effect of Extended Aging and Oxidation
on Linear Poly­(propylenimine)-Mesoporous Silica Composites for CO_2_ Capture from Simulated Air and Flue Gas Streams. Acs Appl. Mater. Interfaces.

[25] Jahandar
Lashaki M., Khiavi S., Sayari A. (2019). Stability of amine-functionalized
CO_2_ adsorbents: a multifaceted puzzle. Chem. Soc. Rev..

[26] Thakkar H. V., Ruba A. J., Matteson J. A., Dugas M. P., Singh R. P. (2024). Accelerated
Testing of PEI-Silica Sorbent Pellets for Direct Air Capture. Acs Omega.

[27] Heydari-Gorji A., Sayari A. (2012). Thermal, Oxidative,
and CO_2_-Induced Degradation
of Supported Polyethylenimine Adsorbents. Ind.
Eng. Chem. Res..

[28] Sayari A., Heydari-Gorji A., Yang Y. (2012). CO_2_-Induced Degradation
of Amine-Containing Adsorbents: Reaction Products and Pathways. J. Am. Chem. Soc..

[29] Li K. M., Jiang J. G., Chen X. J., Gao Y. C., Yan F., Tian S. C. (2016). Research on Urea
Linkages Formation of Amine Functional
Adsorbents During CO_2_ Capture Process: Two Key Factors
Analysis, Temperature and Moisture. J. Phys.
Chem. C.

[30] Didas S. A., Zhu R. S., Brunelli N. A., Sholl D. S., Jones C. W. (2014). Thermal,
Oxidative and CO_2_ Induced Degradation of Primary Amines
Used for CO_2_ Capture: Effect of Alkyl Linker on Stability. J. Phys. Chem. C.

[31] Vu Q. T., Yamada H., Yogo K. (2019). Inhibitors
of Oxidative Degradation
of Polyamine-Modified Silica Sorbents for CO_2_ Capture. Ind. Eng. Chem. Res..

[32] Priyadarshini P., Rim G., Rosu C., Song M., Jones C. W. (2023). Direct Air Capture
of CO_2_ Using Amine/Alumina Sorbents at Cold Temperature. Acs Environ. Au.

[33] Pang S. H., Lee L. C., Sakwa-Novak M. A., Liyely R. P., Jones C. W. (2017). Design
of Aminopolymer Structure to Enhance Performance and Stability of
CO_2_ Sorbents: Poly­(propylenimine) vs Poly­(ethylenimine). J. Am. Chem. Soc..

[34] Pang S. H., Lively R. P., Jones C. W. (2018). Oxidatively-Stable Linear Poly­(propylenimine)-Containing
Adsorbents for CO_2_ Capture from Ultradilute Streams. ChemSusChem.

[35] Al-Absi A. A., Benneker A. M., Mahinpey N. (2024). Amine Sorbents
for Sustainable Direct
Air Capture: Long-Term Stability and Extended Aging Study. Energy Fuels.

[36] Bali S., Chen T. T., Chaikittisilp W., Jones C. W. (2013). Oxidative Stability
of Amino Polymer-Alumina Hybrid Adsorbents for Carbon Dioxide Capture. Energy Fuels.

[37] Vu Q. T., Yamada H., Yogo K. (2021). Effects of
Amine Structures on Oxidative
Degradation of Amine-Functionalized Adsorbents for CO_2_ Capture. Ind. Eng. Chem. Res..

[38] Ahmadalinezhad A., Sayari A. (2014). Oxidative degradation
of silica-supported polyethylenimine
for CO_2_ adsorption: insights into the nature of deactivated
species. Phys. Chem. Chem. Phys..

[39] Ahmadalinezhad A., Tailor R., Sayari A. (2013). Molecular-Level Insights into the
Oxidative Degradation of Grafted Amines. Chem.—Eur.
J..

[40] Bollini P., Choi S., Drese J. H., Jones C. W. (2011). Oxidative Degradation
of Aminosilica Adsorbents Relevant to Postcombustion CO_2_ Capture. Energy Fuels.

[41] Li S., G Y., Calegari Andrade M. F., Hunter-Sellars E., Maiti A., Varni A. J., Tang P., Sievers C., Pang S. H., Jones C. W. (2024). Competing Kinetic
Consequences of
CO_2_ on the Oxidative Degradation of Branched Poly­(ethylenimine). J. Am. Chem. Soc..

[42] Min K., Choi W., Kim C., Choi M. (2018). Oxidation-Stable Amine-containing
Adsorbents for Carbon Dioxide Capture. Nat.
Commun..

[43] Colthup, N. B. ; Daly, L. H. ; Wiberley, S. E. Introduction to Infrared and Raman Spectroscopy; Academic Press, 1990, 339–348.

[44] Srikanth C. S., Chuang S. S. C. (2012). Spectroscopic
Investigation into Oxidative Degradation
of Silica-Supported Amine Sorbents for CO_2_ Capture. ChemSusChem.

[45] Li S. C., Guta Y., Andrade M. F. C., Hunter-Sellars E., Maiti A., Varni A. J., Tang P. C., Sievers C., Pang S. H., Jones C. W. (2024). Competing
Kinetic Consequences of
CO_2_ on the Oxidative Degradation of Branched Poly­(ethylenimine). J. Am. Chem. Soc..

[46] Rim G., Kong F. H., Song M. Y., Rosu C., Priyadarshini P., Lively R. P., Jones C. W. (2022). Sub-Ambient
Temperature Direct Air
Capture of CO_2_ using Amine-Impregnated MIL-101­(Cr) Enables
Ambient Temperature CO2 Recovery. JACS Au.

