# Supplementary material for: Effect of Storage Conditions on Efficacy of Poly(ethylenimine)-Alumina CO2 Sorbents
Source: ACS Omega. 2026 Feb 19;11(8):13405–12. doi: 10.1021/acsomega.5c10742 (PMC12961450; doi:10.1021/acsomega.5c10742)
Supplement: Supplementary file 1 [file ao5c10742_si_001.pdf]

Supporting Information

Effect of Storage Conditions on Efficacy of Poly(ethylenimine)-Alumina CO_2 Sorbents

Yoseph A. Guta,[1] Iman Nezam,[1,A] Juliana Carneiro,[1,B]
Samantha Waters,[1,3] Enerelt Burentugs,[1,4] Carsten Sievers,[1] Christopher W. Jones[1]*

1. *School of Chemical and Biomolecular Engineering, Georgia Institute of Technology, Atlanta, GA 30332, United States*

*Corresponding authors: cjones@chbe.gatech.edu

[A] Current address: Evonik, 7001 Hamilton Blvd, Trexlertown, PA 18087, United States; iman.nezam@evonik.com
[B] Current address: Columbia University, Department of Chemical Engineering, New York, New York 10027, United States; js6441@columbia.edu
[C] Current address: University of Texas at Austin, McKetta Department of Chemical Engineering and Texas Materials Institute, Austin, Texas 78712, United States; sam.waters@utexas.edu
[D] Current address: Center for Catalytic Science and Technology, Department of Chemical and Biomolecular Engineering, University of Delaware, Newark, Delaware 19716, United States; enerelt@udel.edu

Table S1: Pore volume and surface area for bare γ-Al_2O_3, 70% and 100% pore filling PEI/γ-Al_2O_3 sorbents

	Pore volume (cm^3/g)	BET Surface area (m^2/g)	Average Pore Size (nm)
γ-Al_2O_3	0.99	118	31.2
70% Pore fill PEI/ γ-Al_2O_3	0.23	15.3	8.9
100% Pore fill PEI/ γ-Al_2O_3	0.09	0.63	0.7

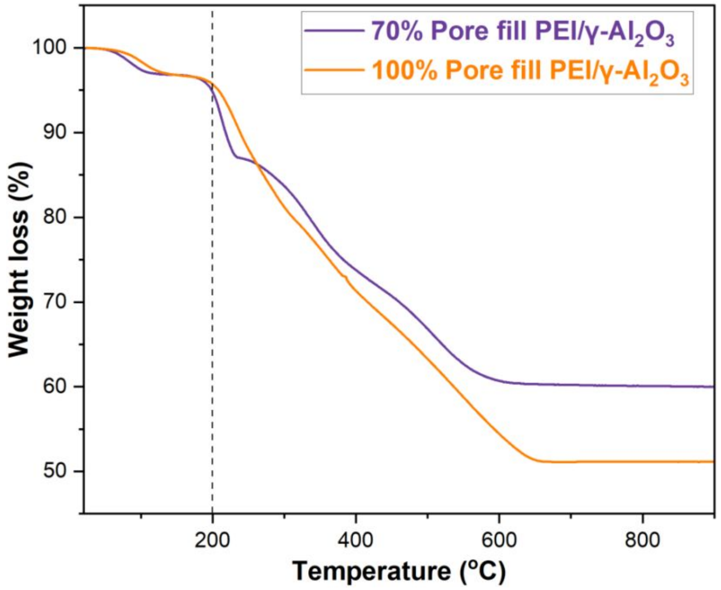

Figure S1: Thermogravimetric combustion of 70% and 100% pore filling PEI/γ-Al_2O_3 sorbents

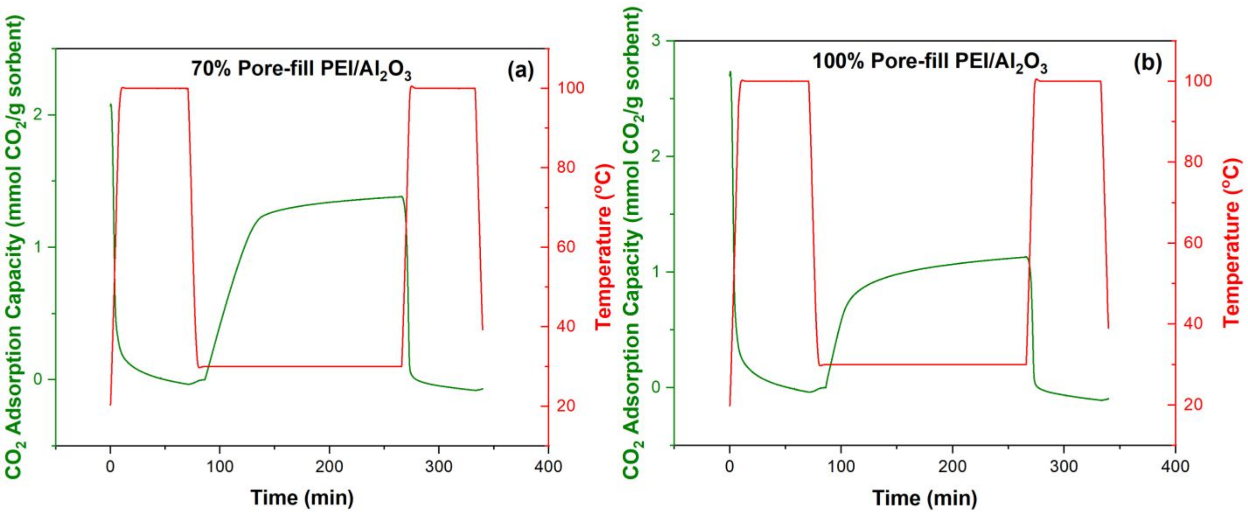

Figure S2: CO_2 adsorption capacity of pristine (a) 70% and (b) 100% pore filling (PF) PEI/γ-Al_2O_3 sorbents at 30 °C

Equation S1: Sorbent deactivation expression to determine CO_2 adsorption capacity loss after a specified time and condition:

$$\text{Sorbent Deactivation} = \frac{CO_2 \text{ capacity Fresh} - CO_2 \text{ capacity Deactivated}}{CO_2 \text{ capacity Fresh}}$$

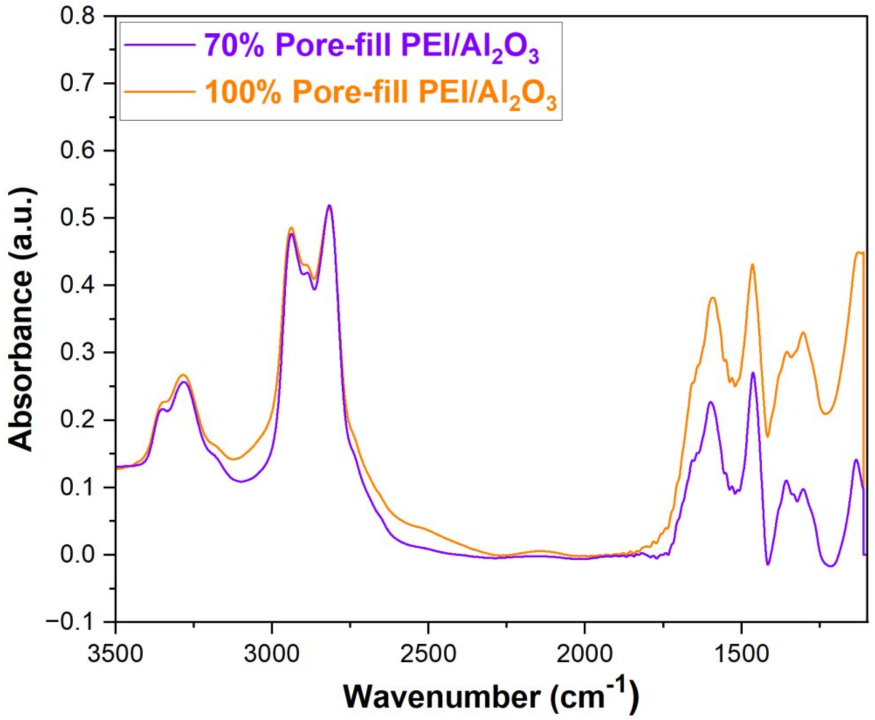

Figure S3: FTIR spectra of pristine 70% and 100% pore fill (PF) PEI/γ-Al_2O_3 sorbent at 30 °C

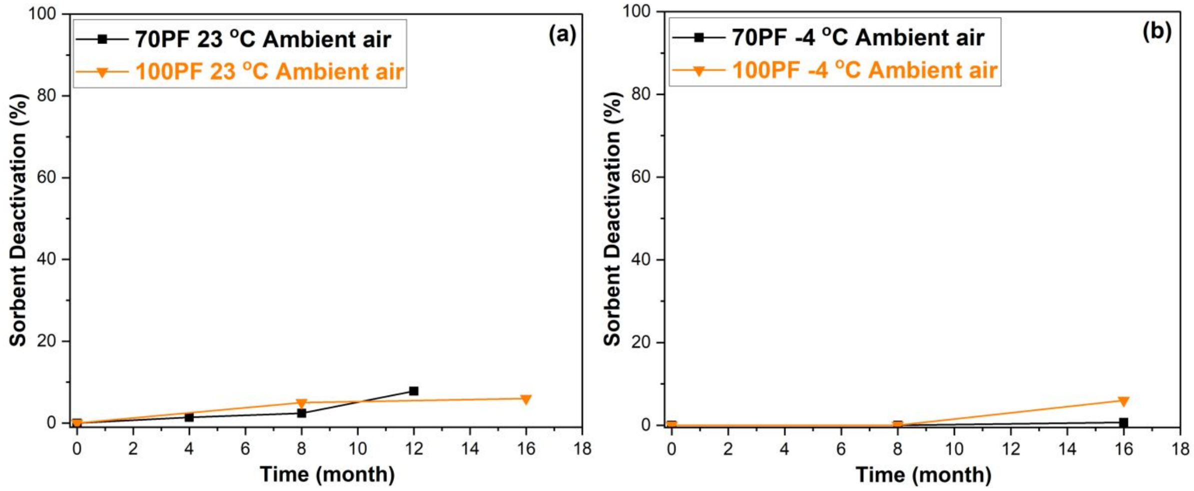

Figure S4: Sorbent deactivation under ambient air for the 70% and 100% pore filling (PF) PEI/γ-Al_2O_3 sorbent aging at (a) 23 °C, and (b) -4 °C

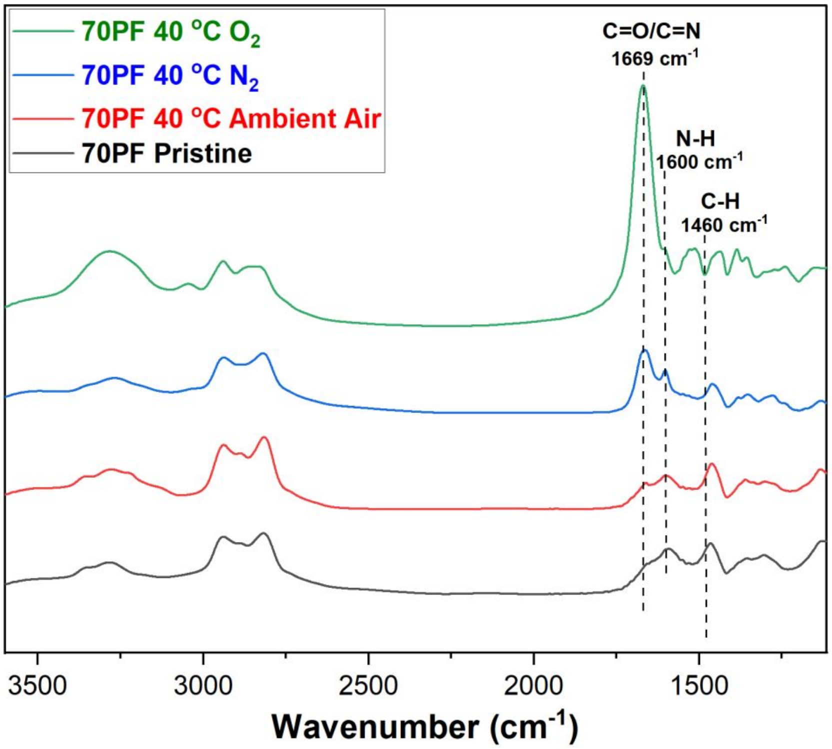

Figure S5: FTIR spectra of 70% pore fill PEI/γ-Al_2O_3 sorbent aged under N_2, ambient air, and O_2 at 40 °C in comparison to pristine sorbent.

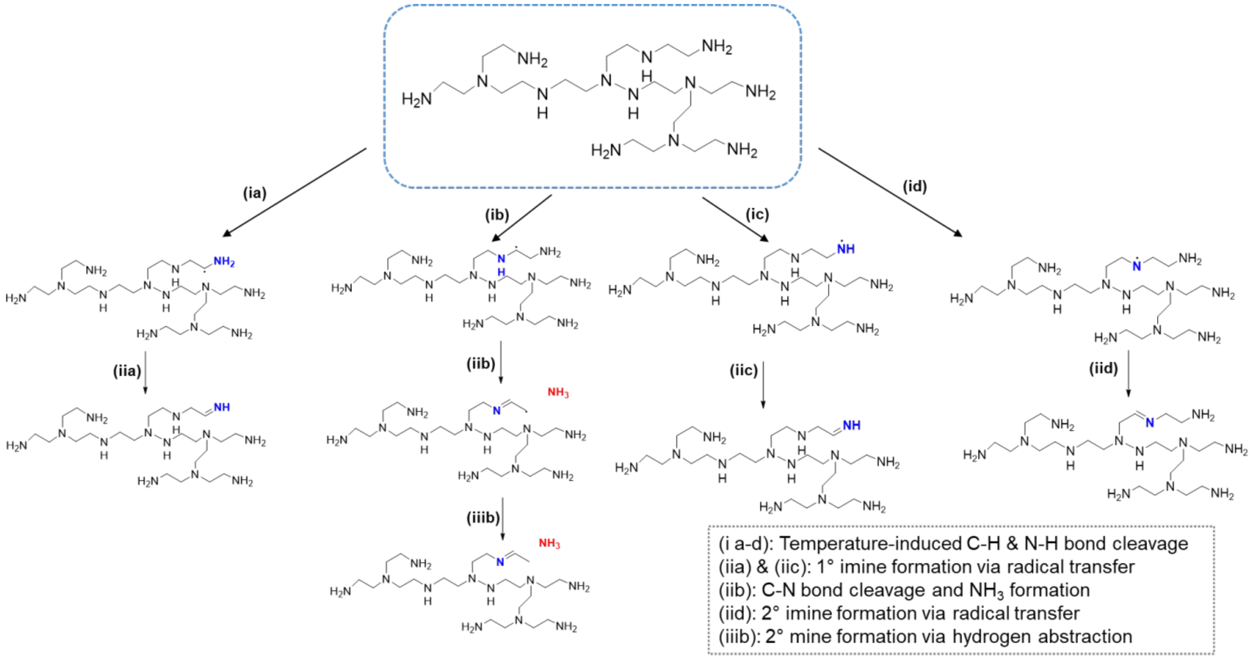

Scheme S1: Examples of non-oxidative thermal degradation reaction pathways[1]

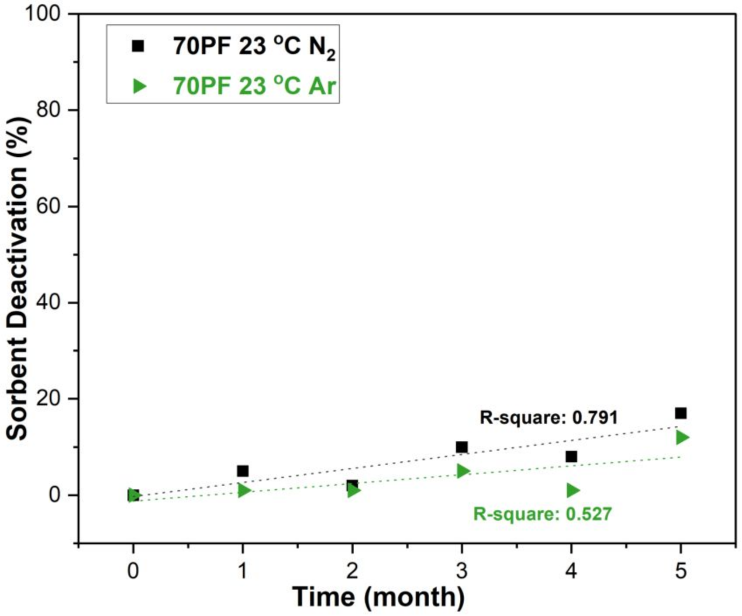

Figure S6: Sorbent deactivation of 70% pore fill (PF) PEI/γ-Al_2O_3 sorbent aged under N_2 and Ar at 23 °C

Table S2: Absolute CO_2 adsorption capacity of each PEI/γ-Al_2O_3 sorbent.
The two values for fresh CO_2 adsorption capacity in some cases are due to the additional experiments conducted.

Time (month)	N_2-40 °C Capacity (mmol CO_2/g sorbent) Fresh (months 1& 2):1.28 Fresh:1.38	N_2-40 °C-N_2 Capacity (mmol CO_2/g sorbent) Fresh:1.28	Air-40 °C Capacity (mmol CO_2/g sorbent) Fresh (months 1& 2):1.28 Fresh (month 4, 8, 12) : 1.38	Air-40 °C-N_2 Capacity (mmol CO_2/g sorbent) Fresh:1.28	Time (month)	O_2-40 °C Capacity (mmol CO_2/g sorbent) Fresh:1.38
1	0.91	0.87	0.68	0.76	4	0.10
2	0.47	0.44	0.47	0.44	8	0.07
4	0.11		0.35		12	0.04
8	0.10		0.32			
12	0.08		0.22			

	100PF-Air-40 °C Capacity (mmol CO_2/g sorbent)		100PF-Air-23 °C Capacity (mmol CO_2/g sorbent)	100PF-Air--4°C Capacity (mmol CO_2/g sorbent)		70PF-Air-23 °C Capacity (mmol CO_2/g sorbent)	70PF-Air--4 °C Capacity (mmol CO_2/g sorbent)

Time (month)	Fresh:1.13	Time (month)	Fresh:1.13	Fresh:1.13	Time (month)	Fresh:1.38	Fresh:1.38
4	0.66	8	1.07	1.13	4	1.36	1.38
8	0.51	16	1.30	1.06	8	1.35	1.38
12	0.47				12	1.27	1.37

Time (month)	N_2-40 °C-Humid Capacity (mmol CO_2/g sorbent) Fresh:1.44	CO_2/N_2-40 °C Capacity (mmol CO_2/g sorbent) Fresh:1.35	CO_2-40 °C Capacity (mmol CO_2/g sorbent) Fresh:1.28	CO_2-40 °C-N_2 Capacity (mmol CO_2/g sorbent) Fresh:1.28	Ar-40 °C Capacity (mmol CO_2/g sorbent) Fresh:1.28	Ar-40 °C-N_2 Capacity (mmol CO_2/g sorbent) Fresh:1.28
1	0.92	1.35	1.28	1.28	0.70	0.69
2	0.37	0.72	1.28	1.28	0.49	0.46
3	0.30	0.61				
4	0.24	0.51				
5	0.20	0.39				

Time (month)	N_2-23 °C Capacity (mmol CO_2/g sorbent) Fresh:1.49	Ar-23 °C Capacity (mmol CO_2/g sorbent) Fresh:1.28	CO_2-23 °C Capacity (mmol CO_2/g sorbent) Fresh:1.28	CO_2-23 °C-N_2 Capacity (mmol CO_2/g sorbent) Fresh:1.28
1	1.42	1.27	1.28	1.28
2	1.46	1.27	1.28	1.28
3	1.34	1.22		
4	1.37	1.27		
5	1.24	1.13		

Reference

(1) Carneiro, J. S. A.; Innocenti, G.; Moon, H. J.; Guta, Y.; Proano, L.; Sievers, C.; Sakwa-Novak, M. A.; Ping, E. W.; Jones, C. W. Insights into the Oxidative Degradation Mechanism of Solid Amine Sorbents for CO2 Capture from Air: Roles of Atmospheric Water. *Angew Chem Int Edit* **2023**. DOI: 10.1002/anie.202302887.
